# Effects of Quercetin on Tubular Cell Apoptosis and Kidney Damage in Rats Induced by Titanium Dioxide Nanoparticles

**DOI:** 10.21315/mjms2018.25.2.8

**Published:** 2018-04-27

**Authors:** Hadis Alidadi, Layasadat Khorsandi, Maryam Shirani

**Affiliations:** 1 Student Research committee, Ahvaz Jundishapur University of Medical Sciences, Ahvaz, Iran; 2 Cell & Molecular Research Center, Faculty of Medicine, Ahvaz Jundishapur University of Medical Sciences, Ahvaz, Iran; 3 Department of Toxicology, Faculty of Pharmacy, Ahvaz Jundishapur University of Medical Sciences, Ahvaz, Iran

**Keywords:** nanoparticles, quercetin, nephrotoxicity, oxidative stress, anti-oxidants, rats

## Abstract

**Background:**

Recent studies have demonstrated that many nanoparticles have an adverse or toxic effect on the kidney.

**Objective:**

To investigate the nephroprotective effect of *quercetin* (QT) against renal injury induced by titanium dioxide nanoparticles (NTiO2) in rats.

**Methods:**

NTiO_2_-intoxicated rats received 50 mg/kg of NTiO_2_ for seven days. The QT *+* NTiO_2_ group was pretreated with QT for seven days before being administered NTiO_2_. Uric acid, creatinine, and blood urea nitrogen were considered to be biomarkers of nephrotoxicity. Catalase (CAT) and superoxide dismutase (SOD) activities and renal levels of malondialdehyde (MDA) were measured to assess the oxidative stress caused by NTiO_2_.

**Results:**

NTiO_2_ significantly increased the plasma level of the biomarkers. It also significantly decreased the activities of CAT (*P =* 0.008) and SOD (*P =* 0.004), and significantly increased the MDA levels (*P =* 0.007). NTiO_2_ caused proximal tubule damage, the accumulation of red blood cells, the infiltration of inflammatory cells, and reduced the glomerular diameters, as well as induced apoptosis in the proximal tubules. Pre-treatment with QT attenuated the histological changes, normalised the plasma biomarkers, suppressed oxidative stress, ameliorated the activities of CAT (*P =* 0.007) and SOD (*P =* 0.006), and reduced apoptosis (*P <* 0.001).

**Conclusion:**

QT was found to have a potent protective effect against nephrotoxicity induced by NTiO_2_ in rats. It also reduced apoptosis caused by NTiO_2_.

## Introduction

The kidney is one of the most sensitive organs to toxic substances in the body because of its high blood flow and its ability to concentrate waste (1). Previous studies demonstrated that the administration of nanoparticles (NPs) to rodents resulted in the particles accumulating in various tissues, including the liver, brain, kidney, and spleen (2). An NP is a microscopic particle that has at least one dimension that is less than 100 nm. Toxicological studies have confirmed that NPs are potentially harmful because of their high surface area to volume ratio and unique physicochemical properties (3). Among the various metal nanomaterials, titanium dioxide nanoparticles (NTiO_2_) are used in a variety of consumer products, including sunscreens, cosmetics, clothing, electronics, paints, and surface coatings (4). NTiO_2_ is also widely used in toothpaste, food colourants, and nutritional supplements. However, NTiO_2_ can accumulate in the renal tissue and induce renal injuries (5).

In recent years, herbal medicines, such as flavonoids, have been shown to have protective effects against chemically induced toxicities (6). For example, quercetin (QT) is a powerful flavonoid that has a wide range of health benefits because of its anti-hypertensive, anti-diabetic, anti-inflammatory, anti-oxidant, anti-cancer, neuroprotective, and anti-viral activities (7). QT is found in lettuce, onion, tomato, red grapes, olive oil, apple skin, tea, coffee, bracken fern, and citrus fruits (8). QT also has a protective effect against some toxic materials, such as arsenic, bisphenol A, and lead, in kidney tissue (9–11). QT has anti-apoptotic effects against some drugs and chemicals, such as doxorubicin, cyclophosphamide, and lead (11, 12). In the present work, the effect of QT on NTiO_2_-induced nephrotoxicity and apoptosis in rats was investigated.

## Materials and Methods

### Animals

Thirty-two healthy adult female Wistar rats, 8–10 weeks old and 180 g–200 g, were used in this work. The estrous cycle was not synchronised in the rats. The rats were purchased from the Experimental Research Center of Ahvaz Jundishapur University of Medical Sciences. The rats were maintained in individual cages and kept under a light–dark cycle of 12:12 at a temperature of 22 °C ± 3 °C and a humidity of 50% ± 5%. They were also given free access to commercial food (pellets) and water.

This work was performed according to the guidelines of the Animal Ethics Committee of Ahvaz Jundishapur University of Medical Sciences (approval number: IR.AJUMS. REC.1395.650).

### Experimental Design

The study was based on the block randomisation method. Before the experiment, the 32 rats were divided into four groups. Based on the power calculation and estimation of attrition from our pilot study and previous studies (6, 13), the sample size was determined to be eight rats per group. The control group was given saline for three weeks. The QT group received 75 mg/kg QT for three weeks (14). The NTiO_2_-intoxicated group received 50 mg/kg NTiO_2_ on day 7 after a daily administration of saline and was followed for two weeks. The QT + NTiO_2_ group received 75 mg/kg QT for one week followed by the concomitant administration of 50 mg/kg NTiO_2_ for two weeks. All treatments were given by gavage. The doses of NTiO_2_ and QT were determined based on our pilot study and previous studies (13, 14). The stock solution of NTiO_2_ (Sigma-Aldrich) was prepared in Milli-Q water as previously described (6). The particle size and morphology of the prepared NTiO_2_ was determined using an atomic force microscope (AFM). The NPs had a spherical morphology, homogeneous particle size distribution, and a mean size less than 100 nm.

Twenty-four hours after the last administration, blood samples were collected, the rats were euthanised, and the renal tissues were removed and weighed. The left kidneys were maintained in a freezer at −80 °C to assess the superoxide dismutase (SOD) and catalase (CAT) activities and malondialdehyde (MDA levels. The right kidneys were fixed in 10% formalin for the histological assessments.

### Biochemical Tests

Blood samples from the tail vein of the rats were collected and centrifuged. The plasma concentrations of uric acid, creatinine (Cr), and blood urea nitrogen (BUN) were determined spectrophotometrically using suitable kits.

### Lipid Peroxidation

Lipid peroxidation was measured as previously described (15). Briefly, kidney tissue homogenates were prepared and 500 μL of supernatant from each sample was added to 1.5 mL 10% trichloroacetic acid and centrifuged for 15 min at 5000× g. Then, 1.5 mL of the supernatant was mixed with 2 mL 0.67% Thiobarbituric acid (TBA) and boiled for 0.5 h. After cooling, 2 mL n-butanol was added to each sample and centrifuged at 5000× g for 20 min. The absorbance was read at 535 nm using a spectrophotometer.

### Superoxide Dismutase (SOD) Activity

The SOD activity was determined using a Ransod kit (Randox Laboratories Ltd., UK) as previously described (15). This method produces a water-soluble formazan dye upon reduction with the superoxide anion. The rate of the reduction is linearly related to the xanthine oxidase (XO) activity, which is inhibited by SOD. The inhibition activity of SOD can be measured using a spectrophotometer at 505 nm.

### CAT Activity

CAT activity was measured as described (16) in a previous study. Briefly, the CAT present in the sample reacts with hydrogen peroxide (H_2_O_2_) to generate H_2_O and O_2_. The unconverted H_2_O_2_ can be determined colorimetrically at the optical density (OD) of 570 nm.

### Histological Changes

In this study, six sections per rat were stained with hematoxylin and eosin (H & E) and assessed for the following histological criteria: nuclear pyknosis, infiltration of inflammatory cells, brush border loss, and accumulation of red blood cells (RBC). The average percentage of each feature was determined. The infiltration of inflammatory cells and the accumulation of RBCs were divided into four categories and the averages were considered. The categories were normal (0), weak (1), moderate (2), and intense (3). Two researchers, who were blinded to the control and experimental groups, analysed the slides independently (17).

### TUNEL Staining

The in situ Cell Death Detection kit (POD, Roche) was used for TUNEL (terminal deoxynucleotidyl transferase dUTP nick end labeling) staining. The paraffin sections were dewaxed and incubated with proteinase K for 0.5 h at 24 °C. The sections were exposed to the TUNEL reaction mixture in a humidity chamber at 37 °C for 1 h. The sections were first incubated with Anti-Fluorescein-AP for 0.5 h at 37 °C, rinsed in deionised water, and incubated with DAB substrate (Sigma-Aldrich) for 5 min. Cells with a homogeneous dark brown nucleus were considered to be TUNEL-positive cells (18).

### Statistical Analysis

To make comparisons between the four groups, a statistical analysis was performed using one-way ANOVA followed by a post hoc least significant difference (LSD) or Tukey’s test for multiple pairwise comparisons. Data were expressed as the mean and standard deviation (SD). A *P*-value less than 0.05 was considered statistically significant.

## Results

### Body and Renal Weights

The body weights in the control and experimental groups were similar and there were no significant differences between them. The body and renal weights in the NTiO_2_-intoxicated group significantly decreased (*P* = 0.009 and *P* = 0.006, respectively). The QT-pretreated group had significantly higher body and renal weights compared with the NTiO_2_-intoxicated group (*P* = 0.042 and *P* = 0.036, respectively). The results are shown in Table 1.

### Biochemical Tests

**Figure 1:**
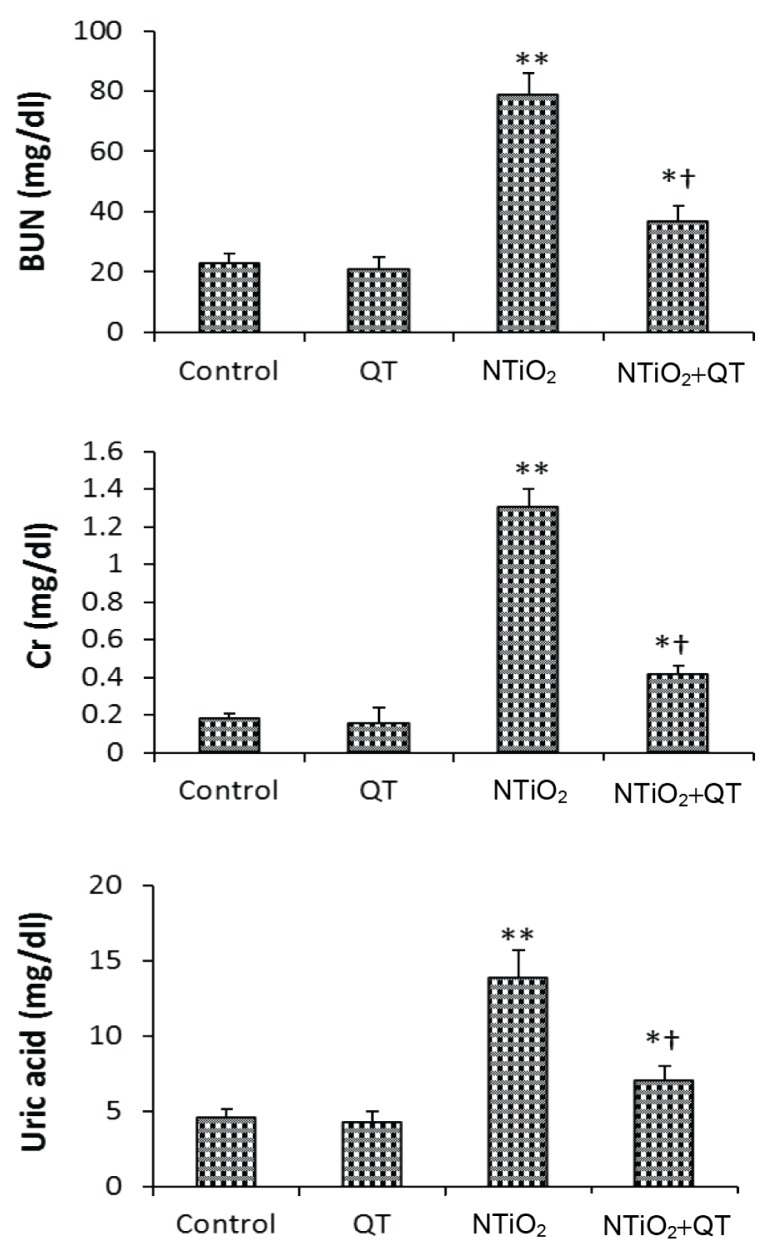
Biochemical tests of control and experimental groups. Values expressed as mean ± SD for eight rats. * *P* < 0.05, ** *P* < 0.001, † *P* < 0.01; * and † symbols respectively indicate comparison to control and NTiO2-intoxicated groups

In the QT group, the blood levels of uric acid, Cr, and BUN were slightly decreased. However, the blood concentrations of uric acid, Cr, and BUN were significantly elevated in the NTiO_2_ group (*P* < 0.001). In the QT + NTiO_2_ group, the levels of uric acid, Cr, and BUN were significantly reduced compared with the NTiO_2_-intoxicated group (*P* = 0.006, *P* = 0.003, and *P* = 0.007, respectively). The results are shown in Figure 1.

### MDA Level, and SOD and CAT Activities

**Figure 2:**
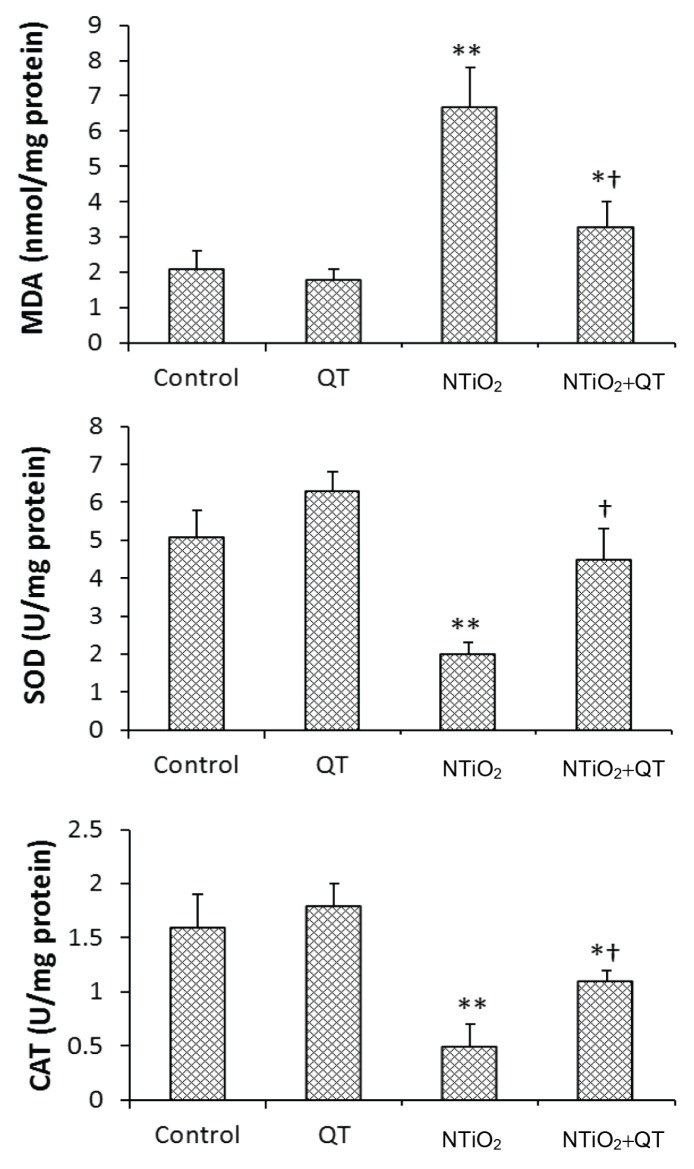
MDA level, SOD and CAT activities of control and experimental groups. Values are expressed as mean ± SD for eight rats. *P < 0.05, ** *P* < 0.01, † *P* < 0.01; * and † symbols respectively indicate comparison to control and NTiO2-intoxicated groups

NTiO_2_ significantly increased the renal level of MDA (*P* = 0.007). In the QT + NTiO_2_ group, the renal level of MDA was significantly decreased compared with the NTiO_2_-intoxicated group (*P* = 0.08). The CAT and SOD activities were significantly reduced in the NTiO_2_ group (*P* = 0.008 and *P* = 0.004, respectively). The CAT and SOD activities were significantly elevated in the QT-pretreated group compared with the NTiO_2_-intoxicated group (*P* = 0.006 and *P* = 0.007, respectively). The results are shown in Figure 2.

### Histological Changes

**Figure 3:**
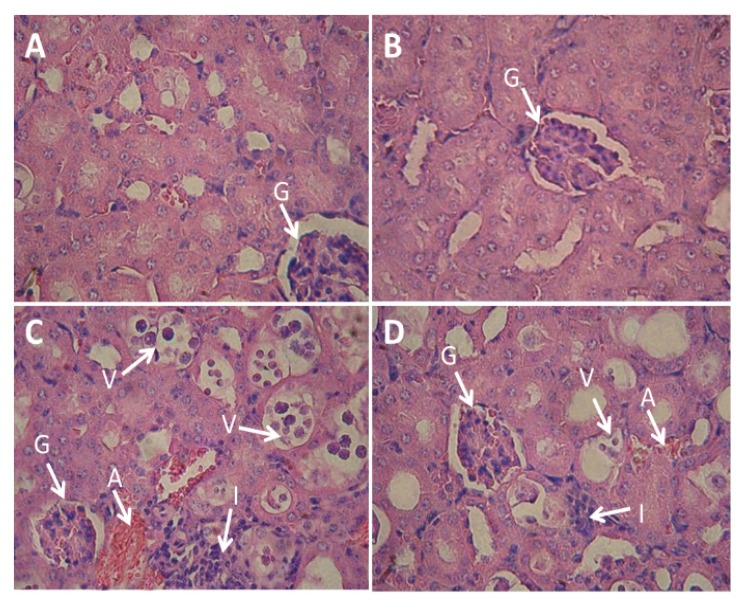
Light microscopy of cross sections of H & E stained testis from control and experimental groups. A: Control group; B: QT group; C: NTiO2-intoxicated group; D: QT + NTiO2 group. A: accumulation of RBCs, G: glomerulus, I: infiltration of inflammatory cells, V: vacuolisation in proximal tubules: Magnifications: ×400

The kidney sections in both the control and QT groups all had a normal appearance. The administration of NTiO_2_ significantly increased the infiltration of inflammatory cells, accumulation of RBCs, brush border loss, and fat deposits in the proximal cells, while the diameter of the glomerulus was significantly decreased. The administration of QT + NTiO_2_ ameliorated the proximal tubule damage, infiltration of inflammatory cells, accumulation of RBCs, and the diameter of the glomerulus compared with the NTiO_2_-intoxicated group (Figure 3 and Table 2).

### TUNEL Staining

**Figure 4:**
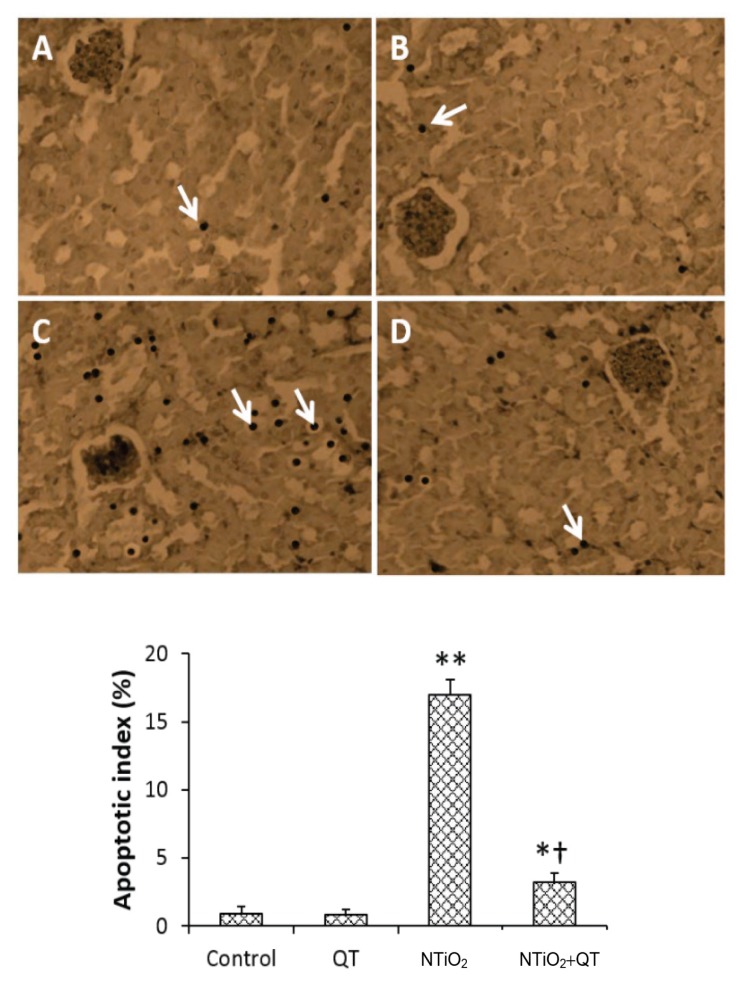
TUNEL staining in the kidney cross sections (Magnifications: ×250) and apoptotic index. A: Control group; B: QT group; C: NTiO2-intoxicated group; D: QT + NTiO2 group. Arrows indicate apoptosis (TUNEL positive cells) in the proximal cells. Values expressed as mean ± SD for 8 rats. *P < 0.05, **P < 0.001, † *P* < 0.001; * and † symbols respectively indicate comparison to control and NTiO2-intoxicated groups

*Apoptosis was observed only* in the proximal cells. As shown in Figure 4, a few proximal cells in some of the tubules in the control and QT groups showed TUNEL-positive staining. In the NTiO_2_-intoxicated group, TUNEL-positive cells were observed in most of the proximal tubules, while the percentage of apoptotic cells was significantly increased (*P* < 0.001). In the QT + NTiO_2_ group, the percentage of TUNEL-positive cells was significantly less than in the NTiO_2_ group (*P* < 0.001).

## Discussion

The results of our study demonstrated that QT protected against kidney damage induced by NTiO_2_. The results showed that the weight of the kidneys decreased in the NTiO_2_ group. This change may have been the result of damage to the proximal tubule and the glomerulus. However, the body weight of the NTiO_2_-intoxicated group also decreased. This suggested that NTiO_2_ has a toxic effect on the kidney and may induce apoptosis in other tissues. Hong and Zhang showed that NTiO_2_ induced liver damage and enhanced the apoptotic index of hepatocytes in rats (19). Another study found that NTiO_2_ can damage mouse testicular tissue and induce germ cell apoptosis (14). Zhao et al. showed that NTiO_2_ induced follicular atresia in female mice (20).

In this study, QT reversed the renal weight loss induced by NTiO_2_. Sangai et al. showed that QT mitigated the effects of bisphenol A on the body and organ weights of mice (10).

One study showed that zinc oxide NP (ZNP) significantly elevated the levels of BUN, Cr, and uric acid biomarkers. When the kidney is damaged, these biomarkers, which are inside the proximal cells of the nephrons, are released into the bloodstream. Hence, elevated concentrations of the biomarkers indicate damage to the proximal cells (21). QT caused the levels of the biomarkers to decrease, which indicates that this flavonoid has beneficial effects on the proximal cells.

This study showed that NTiO_2_ has necrotic effects on the kidney as evidenced by the destruction of tubular structure, vacuolation (fat deposit), nuclear pyknosis of the proximal cells, and the accumulation of RBCs and inflammatory cells. In a study by Fartkhooni et al., the administration of NTiO_2_ induced damage to the proximal tubules and glomerulus (22). In this study, histological changes that were caused by NTiO_2_ were significantly attenuated by QT. The improvement of renal tissue was accompanied by a significant reduction in the plasma levels of the biomarkers and a significant increase in renal weight.

Thus, due to the anti-inflammatory and anti-oxidant properties of QT, it may inhibit NTiO_2_-induced renal damage in rats. Liu et al. showed that QT also suppressed the inflammatory response induced by lead in the kidneys of rats (23).

In this study, the MDA concentration in kidney tissue was significantly increased by NTiO_2_. MDA is an indicator of the peroxidation process. Fat deposits in the proximal cells indicate abnormal fat metabolism. Previous studies demonstrated that NTiO_2_ induced lipid peroxidation (fat deposit) and oxidative stress in the kidney of rodents (24). Another study found that NTiO_2_ generates apoptosis and oxidative stress in human nephrons (25). Zhao et al. suggested that NTiO_2_ causes the accumulation of reactive oxygen species, suppresses the anti-oxidative systems, and triggers nephritis in the kidney (26).

As discussed in the results, QT attenuated the fat deposits in proximal cells induced by NTiO_2_. Another study found that QT improved the fat deposits in the proximal tubules of doxorubicin-intoxicated rats (27).

In this study, the significantly reduced activities of SOD and CAT indicated that NTiO_2_ induced oxidative stress in renal tissue. However, pre-treatment with QT significantly reversed the activities of CAT and SOD, which indicated the anti-oxidant property of *QT.*

In this study, both apoptosis and necrosis were observed at the same time in the NTiO_2_ group. Wilhelmi et al. demonstrated that ZNP induced both apoptosis and necrosis in macrophages (28). Liu et al. showed that QT inhibited apoptosis and DNA damage induced by lead in the kidney of rats (11). We also observed that NTiO_2_ increased apoptosis in the proximal cells. Pre-treatment with QT effectively decreased the apoptotic index in kidney tissue. Özyurt et al. showed that QT prevented apoptosis in the kidney tissue induced by radiation (29). It has also been reported that QT protects the kidney of mice against oxidative stress induced by arsenic (9).

The beneficial effects of QT on nephrotoxicity have also been reported by other researchers. For example, Faddah et al. showed that QT reduced nephrotoxicity induced by ZNP in rats (30). QT also reduced cisplatin-induced nephrotoxicity in rats (31), and was found to attenuate the damage to kidneys caused by pesticides (32).

## Conclusion

This study showed that QT prevents NTiO_2_-induced apoptosis and nephrotoxicity in female rats. QT protected kidney cells due to its antioxidant and anti-apoptotic effects. Future research should be done to confirm the anti-apoptotic effects of QT against NPs in animals.

## Figures and Tables

**Table 1:** Kidney and body weight for control and experimental groups

Groups	Body weight	Kidney weight
Control	235.2 (13.2)	1.03 (0.11)
QT	236.1 (14.2) *P* = 0.174	1.04 (0.13) *P* = 0.223
NTiO_2_	191.8 (15.4) **P = 0.009	0.68 (0.06) **P = 0.006
QT+ NTiO_2_	210.6 (11.5) *P = 0.034, †*P* = 0.042	0.82 (0.07) *P = 0.015, †*P* = 0.036

Values expressed as mean (SD) for eight rats.

* *P* < 0.05,

** *P* < 0.01,

† *P* < 0.05;

* and † symbols respectively indicate comparison to control and NTiO_2_-intoxicated groups.

**Table 2:** Histology assessments in control and experimental groups

Histological criteria	Control	QT	NTiO_2_	QT+ NTiO_2_
Normal (%)	97.7 (3.2)	98.4 (4.7) *P* = 0.123	74.4 (8.2) **P = 0.009	90.3 (11.5) †*P* = 0.007
Tubular vacuolisation (%)	0.00 (0.00)	0.0 (0.00) *P* = 0.63	15.2 (2.2) ***P = 0.000	6.1 (2.6) ***P = 0.000, †*P* = 0.004
Brush border loss (%)	0. 4 (0.06)	0.6 (0.3) *P* = 0.231	4.8 (2.8) **P = 0.002	1.7 (1.5) *P = 0.034, †*P* = 0.005
Infiltration of leukocytes	0.02 (0.004)	0.02 (0.007) *P* = 0.093	2.6 (0.07) ***P = 0.000	0.3 (0.04) **P = 0.004, †*P* = 0.005
Congestion of RBCs	0.14 (0.03)	0.11 (0.04) *P* = 0.081	2.4 (0.04) **P = 0.004	0.2 (0.04) *P = 0.0647, †*P* = 0.006
*Glomerular diameters* (μm)	224 (9.3)	231.7 (7.6) *P* = 0.137	151.9 (6.1) **P = 0.003	197.6 (8.2) *P = 0.059, †*P* = 0.002

Values expressed as mean ± SD for eight rats.

* *P* < 0.05,

** *P* < 0.01,

*** *P* < 0.001,

† *P* < 0.01;

* and † symbols respectively indicate comparison to control and NTiO_2_-intoxicated groups.
